# Take Off, Landing, and Fly Anesthesia

**DOI:** 10.1371/journal.pgen.1003788

**Published:** 2013-09-05

**Authors:** Robert D. Sanders, Mervyn Maze

**Affiliations:** 1 Department of Anaesthesia and Surgical Outcomes Research Centre, University College London Hospital & Wellcome Department of Imaging Neuroscience, University College London, London, United Kingdom; 2 Department of Anesthesia and Perioperative Care, University of California, San Francisco, San Francisco, California, United States of America; The University of North Carolina at Chapel Hill, United States of America

**Figure 1:**
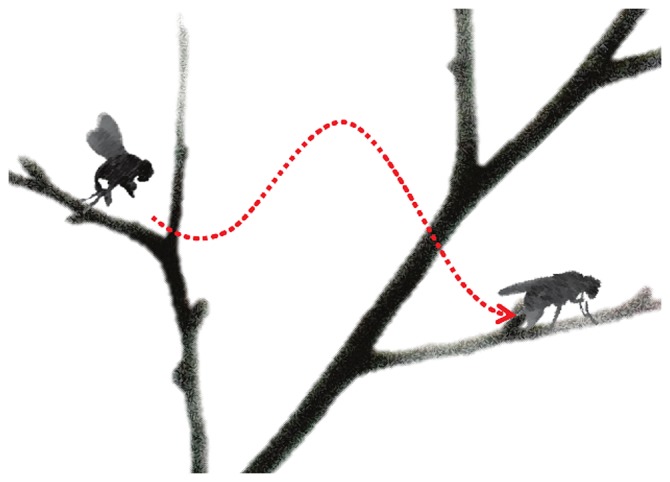
“Take off” and “landing” for fly anesthesia. In this schematic, induction of anesthesia is represented by the fly taking off, with the height of the branch representing the drug dose. “Landing” (or emergence) from anesthesia occurs on a lower branch representing a lower drug dose than “take off”. The difference in height between the branches signifies neural inertia, the resistance in changing between anaesthetized and wakeful states. *The parts of this figure are adapted from images by Antonia Foy (* http://www.flickr.com/photos/antoniafoy/5542985500/ *) and John Tann (* http://www.flickr.com/photos/31031835N08/8112956476/ *;* http://www.flickr.com/photos/31031835N08/5387406710/ *), available on Flickr under a Creative Commons Attribution license*.

Anesthesia can be broken down into several components including unconsciousness, disconnection (unawareness of surgery and/or the environment), unresponsiveness, amnesia, and analgesia (absence of pain) [1]. Monitoring these components within clinical settings is challenging, although unresponsiveness, especially in the absence of neuromuscular blockade, or when a limb has been isolated from generalized paralysis, may be helpful. Unresponsiveness is therefore an appropriate anesthetic endpoint to study in both preclinical and clinical settings as it is well conserved across species and is assessable by observing behavior. While significant progress has been made through studies addressing molecular species [2], neural networks [3], neuroimaging [4], and electroencephalography (EEG) [5], the mechanisms of anesthesia-induced unresponsiveness, and its maintenance, remain elusive [1]. Pioneering work from Dr. Max B. Kelz's laboratory begins to shed light on the mechanisms involved in the induction into (“take off”) and emergence from (“landing”) anesthesia-induced unresponsiveness (referred to now as “anesthesia”; Figure 1).

Classically, anesthetic recovery was considered to involve the reversal of events used in the induction; however, high-resolution EEG data revealed the presence of a hysteresis between the onset and offset of anesthesia that could not be explained in pharmacokinetic terms [5]. An analogy to flying is that landing a plane is not merely the reverse of taking off. Dr. Kelz has characterized the hysteresis as “neural inertia,” envisioning a resistance to change from anesthetized to awake states [6]; studies in his laboratory have demonstrated neural inertia to diverse anesthetic agents in mice and flies [6]–[8]. In mice, the role of the neuromodulators orexin [8] and noradrenaline [7] have been highlighted; in flies, mutation of the Shaker potassium channel [6] led to collapse of neural inertia. From this work the speculation arose that patients who lack neural inertia may be predisposed to anesthesia awareness [6]; others have suggested that increased neural inertia may protect against acute confusion (delirium) on “landing” from anesthesia by preventing patients connecting with their environment too early [1], [9].

In this issue of *PLOS Genetics*, Joiner et al. share their latest insights into the mechanisms of neural inertia based on a series of elegant fly studies [10] that examined both “take off” and “landing” from anesthesia. Four different mutations led to collapsed neural inertia, but two of these four mutations increased *sensitivity* to “take off” of anesthesia while the other two increased *resistance* to “take off” [10]. Furthermore, the differences in mechanisms of induction into, and emergence from, anesthesia were highlighted in studies showing that different mutations targeting glutamatergic signaling exerted different effects on induction and emergence from anesthesia [10]. Differential effects of two volatile anesthetics, halothane and isoflurane, on induction of anesthesia were also noted in some mutants, supporting the notion of discrete mechanisms of anesthesia for individual drugs [10]. Therefore, anesthesia “take off” and “landing” appear to have different neurobiology and henceforth should be considered separately.

Mechanistically, neural inertia is distinguished from arousal by Joiner et al., as hyperaroused mutants did not show altered neural inertia [10]. Further dissociations from arousal were supported by a lack of role for the circadian clock in influencing neural inertia (by studies conducted at different times of day) [10]. An interesting complexity occurs when studying sleep deprivation, however, as sleep-deprived flies showed increased neural inertia relative to rested controls [10]. The overlap between sleep and anesthetic mechanisms has been known for many years [3], but the insight that sleep deprivation did not affect induction of, but rather emergence from, anesthesia is particularly intriguing. While anesthetics converge on the sleep pathway to maintain the anesthetic state [2], [3], direct effects on higher corticothalamic centers (“top-down”) may dictate anesthetic induction [1], [11]. Hence, “take off” may depend on perturbation of corticothalamic activity, while effects on the sleep circuitry may contribute more significantly to the maintenance of, and “landing” from, anesthesia. The work of Joiner et al. adds to seminal discoveries that patients may not “fall asleep” into anesthesia, and whether they “wake up” (analogous to sleep) or emerge remains an exciting question.

Studies that seek to tease apart the neurobiologic correlate of “take off,” as distinct from either maintenance or “landing,” need to consider that passage into and through these states may have induced pharmacodynamics changes akin to tolerance. This problem arises because we cannot simply study arousal mechanisms without first inducing and maintaining the anesthetized state. Long-lasting changes are induced by the administration of general anesthetics [12]; in an analogous manner, changes induced by taking off and flying may affect the ability to land.

When considering the clinical relevance of the data reported by Joiner et al., we need to qualify our enthusiasm. Translating results from invertebrate systems to vertebrates, not to mention surgical patients with co-morbidities, raises several intriguing questions. Both orexin and norepinephrine are important neuromodulators of sleep and anesthesia sensitivity in vertebrates [6]–[8], but are untested in the fly. Mechanistically, it is also unclear how the neurobiology of unresponsiveness in *Drosophila* overlaps with human unresponsiveness [1]. Finally, anesthesia is provided to facilitate surgery, which itself has significant effects that could alter neural inertia (perhaps through noradrenergic effects on connectedness [1]).

Nonetheless, Joiner et al. have provided a new construct in which to consider the state of anesthesia [10]. In aggregate, the work from Kelz's laboratory provides support for their concept of neural inertia, through their careful dissection of the mechanisms of “take off” and “landing” from anesthesia, and for the dissociation of neural inertia from arousal. These findings may presage our understanding of how patients are induced into unresponsiveness, stay unresponsive during anesthesia, and subsequently reanimate. We support the authors' contention that understanding these mechanisms may lead to improvements in anesthesia as well as in conditions involving potentially pathological neural inertia, such as non-drug-induced comatose states. However, more work is required before the impact of these findings alter the perioperative care of surgical patients.
